# Supplementary material for: Label-Free Characterization of Macrophage Polarization Using Raman Spectroscopy
Source: Int J Mol Sci. 2023 Jan 3;24(1):824. doi: 10.3390/ijms24010824 (PMC9821063; doi:10.3390/ijms24010824)
Supplement: Supplementary file 1 [file ijms-24-00824-s001.zip › ijms-2077232-supplementary.pdf]

Supplement for

Label-Free Characterization of Macrophage Polarization Using Raman Spectroscopy

Max Naumann, Natalie Arend, Rustam R. Guliev, Christian Kretzer, Ignacio Rubio, Oliver Werz, Ute Neugebauer

Content

1. Flow cytometry of macrophage populations 3

Supplemental Figure S1: Forward and side scatter density plots (FSC vs. SSC) of detached macrophage phenotypes from the three different donors. 3

2. Fluorescence visualization of macrophage polarization 4

Supplemental Figure S2: 3D image stacks of macrophages of different phenotype 4

Supplemental Figure S3: Fluorescence microscope images of M2 macrophage 4

3. Raman data analysis 5

Supplemental Table S1: Overview of Raman data measured by the Raman imaging system and used for data analysis 5

3.1. N-FINDR false-colour images... 6

Supplemental Figure S4-A. 15 endmembers (most different spectra) found by N-FINDR algorithm and grouped according to corresponding biochemical component.. 6

Supplemental Figure S4-B. False-colour Raman images of M0 macrophages ... 7

Supplemental Figure S4-C. False-colour Raman images of M1 macrophages ... 9

Supplemental Figure S4-D. False-colour Raman images of M2 macrophages . 10

Supplemental Figure S4-E. Bright-field images of M0, M1 and M2 macrophages 11

Supplemental Figure S5. Box-plots of average coefficients (contribution) per pixel split for each donor. 12

Supplemental Figure S6. Raman analysis of extracted lipid spectra. 13

Supplemental Figure S6-A. Averaged spectra of lipids in the fingerprint region extracted from Raman images of different macrophage phenotypes. 13

Supplemental Figure S6-B. Raman band ratios in extracted lipid spectra. Values are split by donor and colored by macrophage phenotype (red: M0, green M1, blue: M2). 14

Supplemental Table S2. Overview of extracted lipid spectra............................ 15

Supplemental Table S3. Raman band ratios of lipid spectra in different macrophage phenotypes. Mean plus-minus standard deviation of each calculated Raman band intensity and Raman band ratio for the extracted lipid spectra shown in Supplemental Figure S6. 15

3.2. PCA-LDA models 16

Supplemental Figure S7-A. Binary PCA-LDA model (trained on averaged spectra) identifying pro-inflammatory M1 macrophages 16

Supplemental Figure S7-B. Dependency of the performance metrics of the binary PCA-LDA model (trained on averaged spectra) discriminating between resting M0 and anti-inflammatory M2 macrophages on the number of principal components used.. 16

Supplemental Figure S7-C. Binary PCA-LDA model (trained on individual spectrum) identifying pro-inflammatory M1 macrophages. 17

1. Flow cytometry of macrophage populations

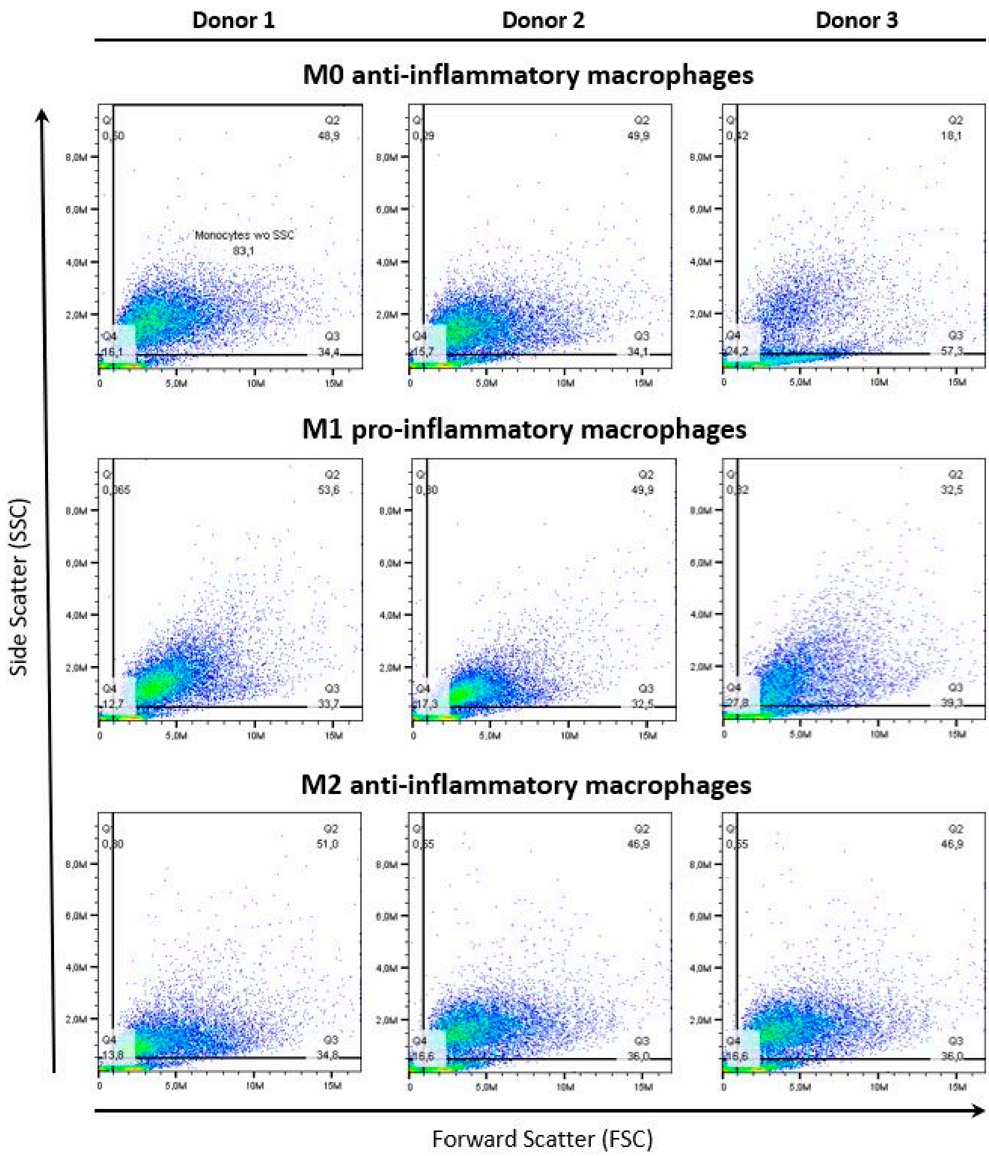

Supplemental Figure S1. Forward and side scatter density plots (FSC vs. SSC) of detached macrophage phenotypes from the three different donors.

Macrophages of M0, M1 and M2 phenotype were detached from their substrates by incubation in PBA-E buffer (PBS, 5mM EDTA, 0.1% sodium azide, 0.5% BSA) for 20 minutes, stained with fluorescence-labelled antibodies and analysed by flow cytometry. Events were gated in quadrants. Only events in the second quadrant (Q2) were used for later analysis of fluorescence. The other quadrants were suspected to be cell debris.

2. Fluorescence visualization of macrophage polarization

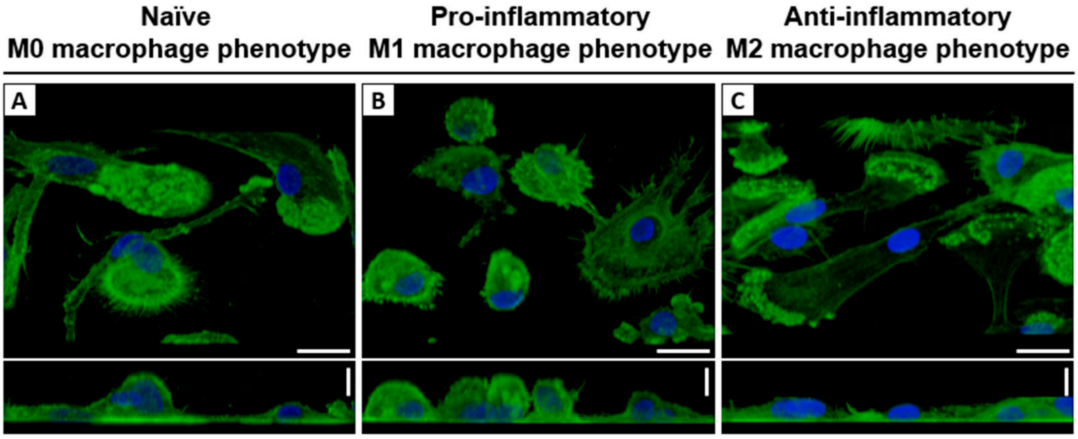

Supplemental Figure S2. 3D image stacks of macrophages of different phenotype. The image shows different macrophage phenotypes with **A:** naïve M0 macrophages, **B:** classically activated M1 macrophages (pro-inflammatory) and **C:** alternatively activated M2 macrophages (anti-inflammatory) from top view (first row) and side view (second row). All samples were stained with DAPI (nucleus, blue) and Phalloidin-AlexaFluor555 (filamentous actin structures, green). Horizontal scale bar = 20 µm, vertical scale bar = 10 µm).

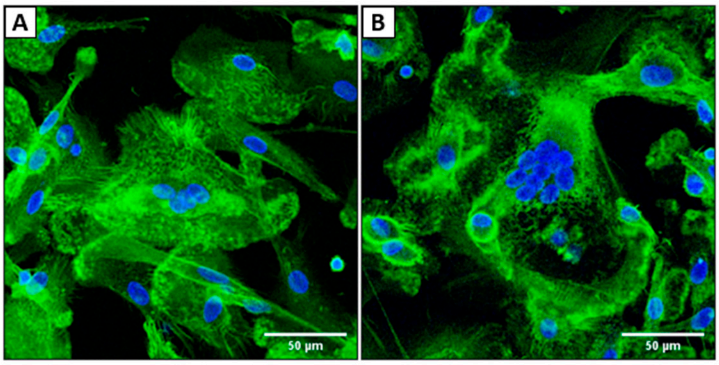

Supplemental Figure S3. Fluorescence microscope images of M2 macrophage: M2 monocyte-derived macrophages exhibited a heterogeneous morphology. Also, the presence of giant multinucleated cells, as depicted here, was occasionally observed (sample from Donor 3).

3. Raman data analysis

Supplemental Table S1. Overview of Raman data measured by the Raman imaging system and used for data analysis. The table indicates the number of measured functional macrophage phenotypes as well as the corresponding number of spectra before and after pre-processing. Removing of non-cellular background accounts for the high reduction of number of spectra after pre-processing.

Group	Number of images recorded				Number of spectra recorded			
	Donor 1	Donor 2	Donor 3	∑	Donor 1	Donor 2	Donor 3	∑
M0	1	6	8	15	4,698	85,147	118,568	208,413
M1	7	10	15	32	17,030	25,000	40,803	82,833
M2	8	7	5	20	59,912	79,950	75,138	215,000
∑	16	23	28	**67**	81,640	190,097	234,509	**506,246**
**Group**	**Number of images after preprocessing**				**Number of spectra after preprocessing**			
	**Donor 1**	**Donor 2**	**Donor 3**	∑	**Donor 1**	**Donor 2**	**Donor 3**	∑
M0	1	6	7	14	2,323	19,280	40,889	62,492
M1	6	10	15	31	6,505	9,862	15,995	32,362
M2	8	7	5	20	29,332	26,641	32,769	88,742
∑	15	23	27	**65**	38,160	55,783	89,653	**183,596**

3.1. N-FINDR false-colour images

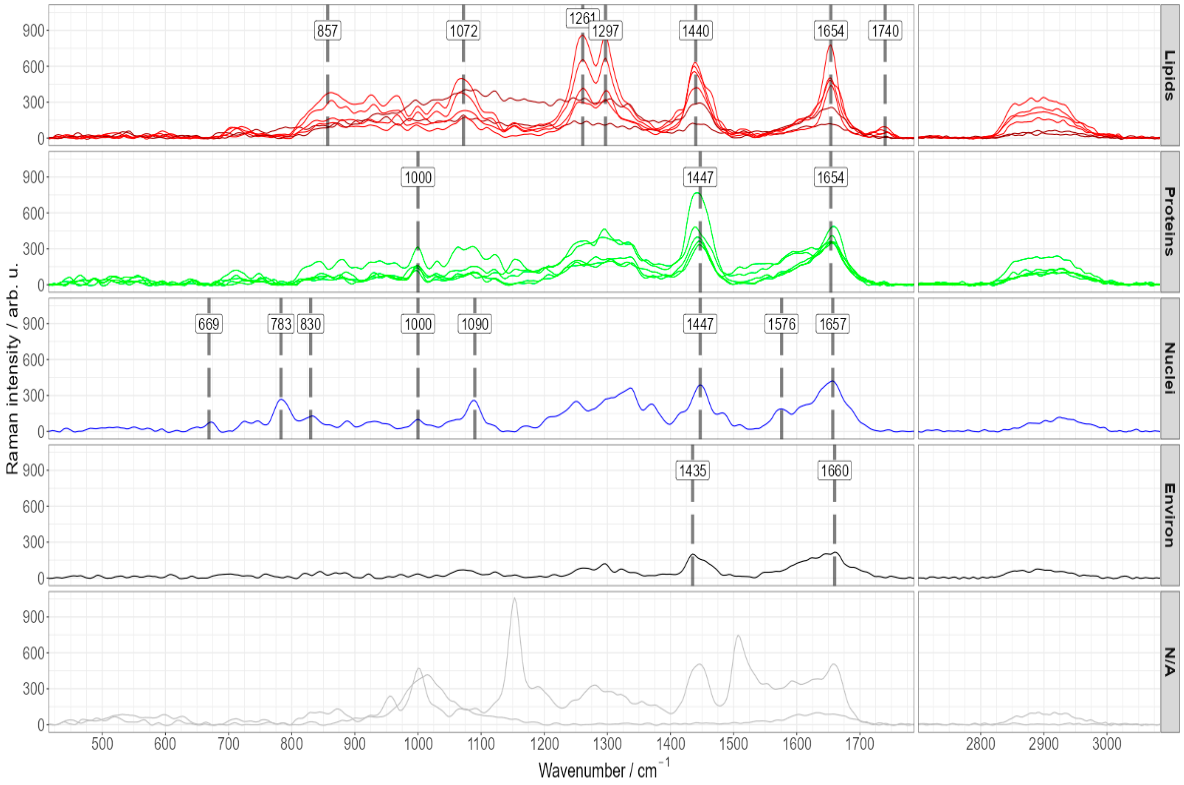

Supplemental Figure S4-A. 15 endmembers (most different spectra) found by N-FINDR algorithm and grouped according to corresponding biochemical component. The spectra are assigned to major biochemical component based on their typical Raman bands. **Red spectra (first row)** represent typical lipid spectra with prominent CH deformation modes at ~1261 cm^{-1} and ~1297 cm^{-1} and C-H stretching vibrations around 2887 cm^{-1}. The sharp bands around 1445 cm^{-1} and 1654 cm^{-1} arise from CH_2/ CH_3 deformation and C=C stretching vibrations, respectively. The band ~1740 cm^{-1} can be assigned to the C=O stretching mode of triacylglycerols. Two spectra (coloured in dark red) were assigned to the lipids although they had a different shape. In that case, the assignment was based on the fact those spectra appeared only in areas of high concentration of lipids. The **green spectra (second row)** have a prominent band at ~1000 cm^{-1} originating from the ring breathing mode of phenylalanine and indicating presence of proteins. Further protein Raman bands are found with the amide vibrational bands (~1654 cm^{-1}) and CH_2 deformation bands (~1447 cm^{-1}). The **blue spectrum (third row)** shows prominent contributions from nucleic acid vibrations, such as DNA and RNA phosphate backbone vibrations at ~787 cm^{-1} and ~1090 cm^{-1} as well as the vibrational modes of DNA bases in the spectral region from ~1255 cm^{-1} to ~1372 cm^{-1}. The **black spectrum (forth row)** shows low overall intensity with spectral contribution of CH-stretching vibrations (~1440 cm^{-1}) and amide I vibrations (~1660 cm^{-1}). These spectra were mainly found at the outer part of the cell where the cell height is decreasing, resulting in less signal. Thus, this endmember has been assigned to cytoplasm/environment. **Grey spectra (last row)** appeared only in a few pixels of 2-3 images and thus were not further assigned.

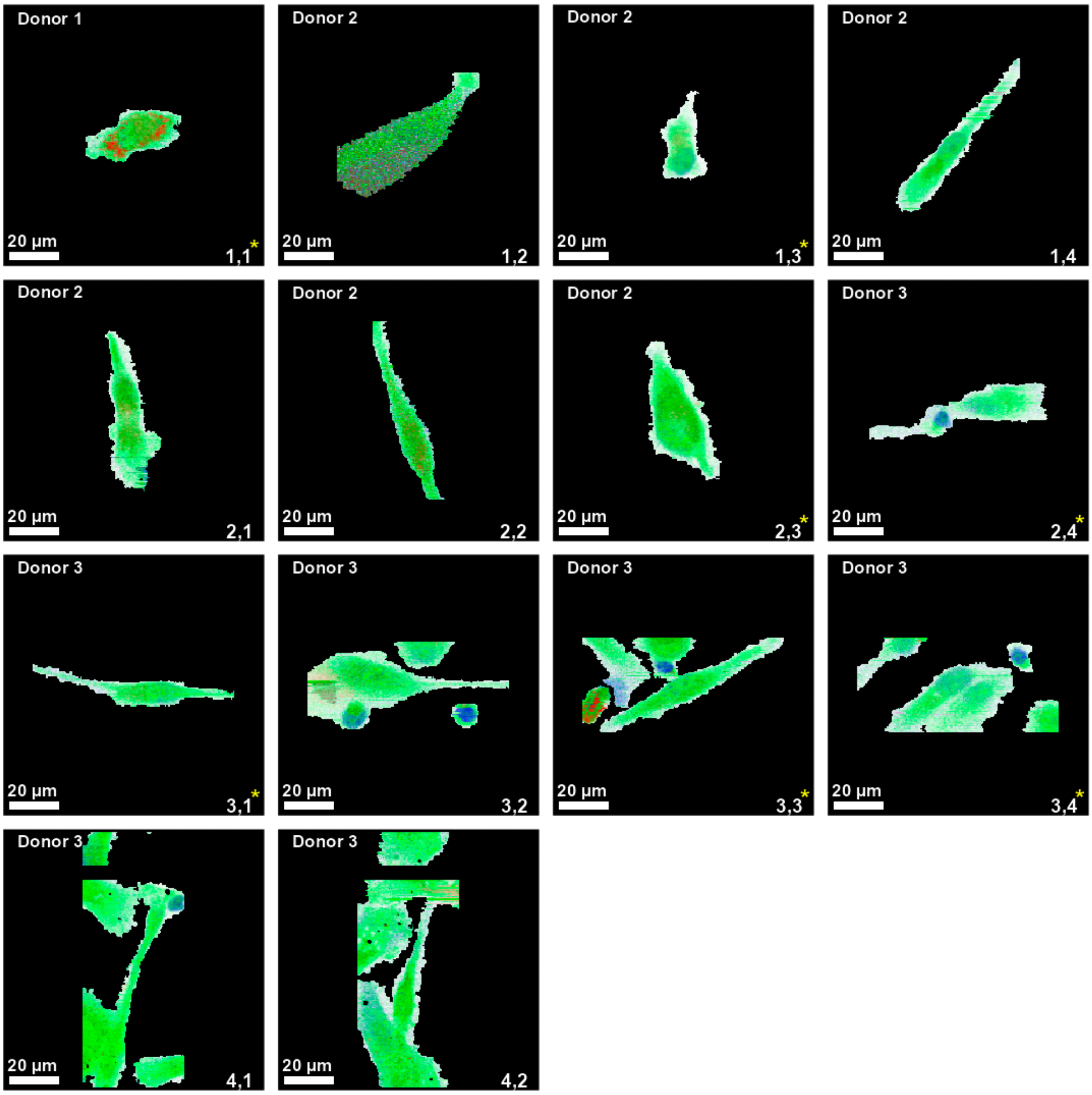

Supplemental Figure S4-B. False-colour Raman images of M0 macrophages. Colours: the colour of a pixel is assigned according to contribution of the corresponding group of endmembers shown in Supplementary Fig. S7-A: red for lipids, green for cytoplasm proteins, blue for nucleus, and white for the cytoplasm/ cell environment (note: this endmember is depicted in black in Fig. S7-A). Scale: To make images comparable, each image is plotted in a square of 110 µm x100 µm area, independent of size of image scan. Scale bar is always 20 µm. Labels: Labels on the top-left indicate donor of the cell; bottom-right labels give a coordinate for easier reference. The images that were selected for finding endmembers have yellow asterisk near the coordinate label.

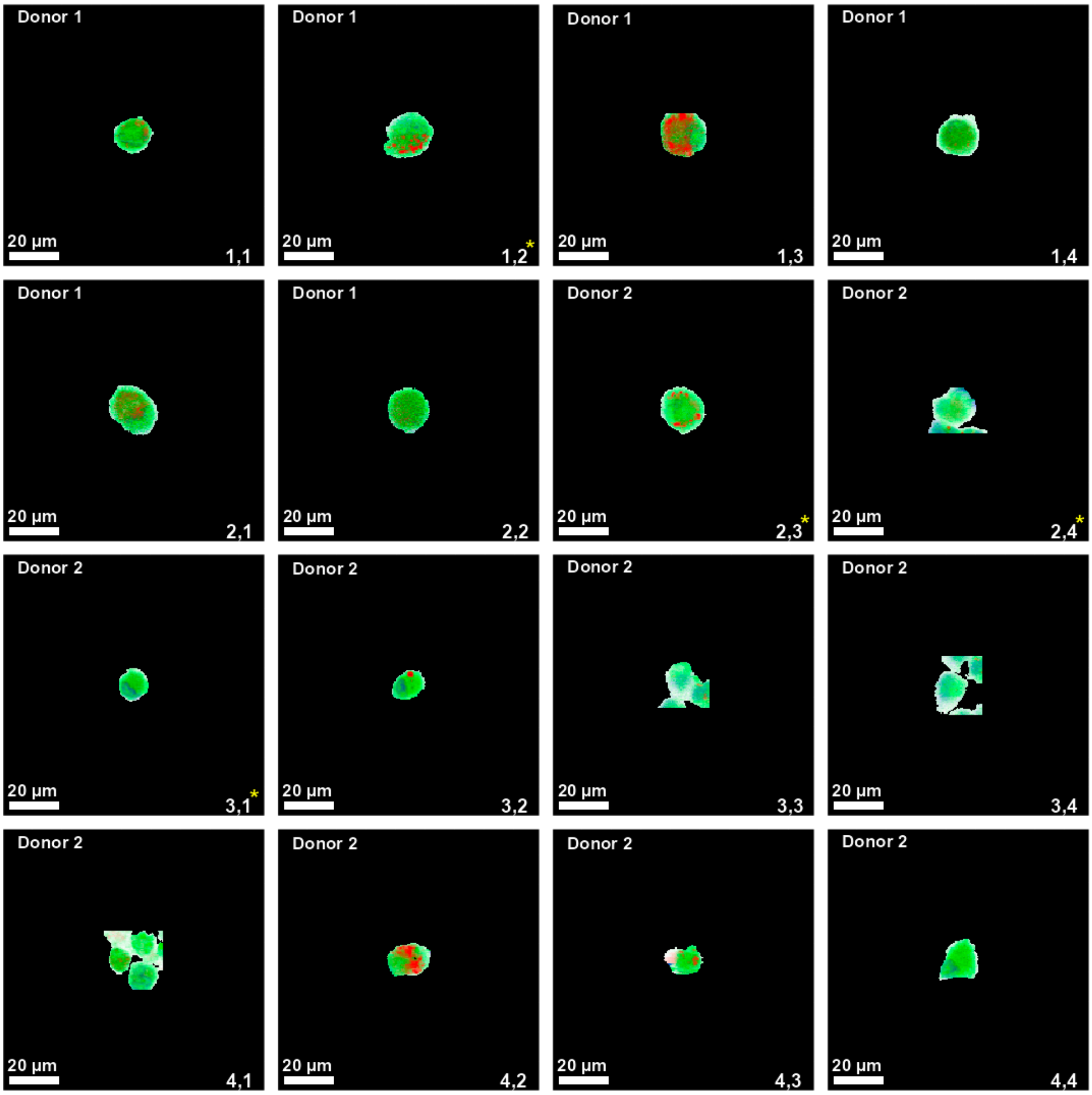
Donor 1
20 µm
1,1
Donor 1
20 µm
1,2*
Donor 1
20 µm
1,3
Donor 1
20 µm
1,4
Donor 1
20 µm
2,1
Donor 1
20 µm
2,2
Donor 2
20 µm
2,3*
Donor 2
20 µm
2,4*
Donor 2
20 µm
3,1*
Donor 2
20 µm
3,2
Donor 2
20 µm
3,3
Donor 2
20 µm
3,4
Donor 2
20 µm
4,1
Donor 2
20 µm
4,2
Donor 2
20 µm
4,3
Donor 2
20 µm
4,4

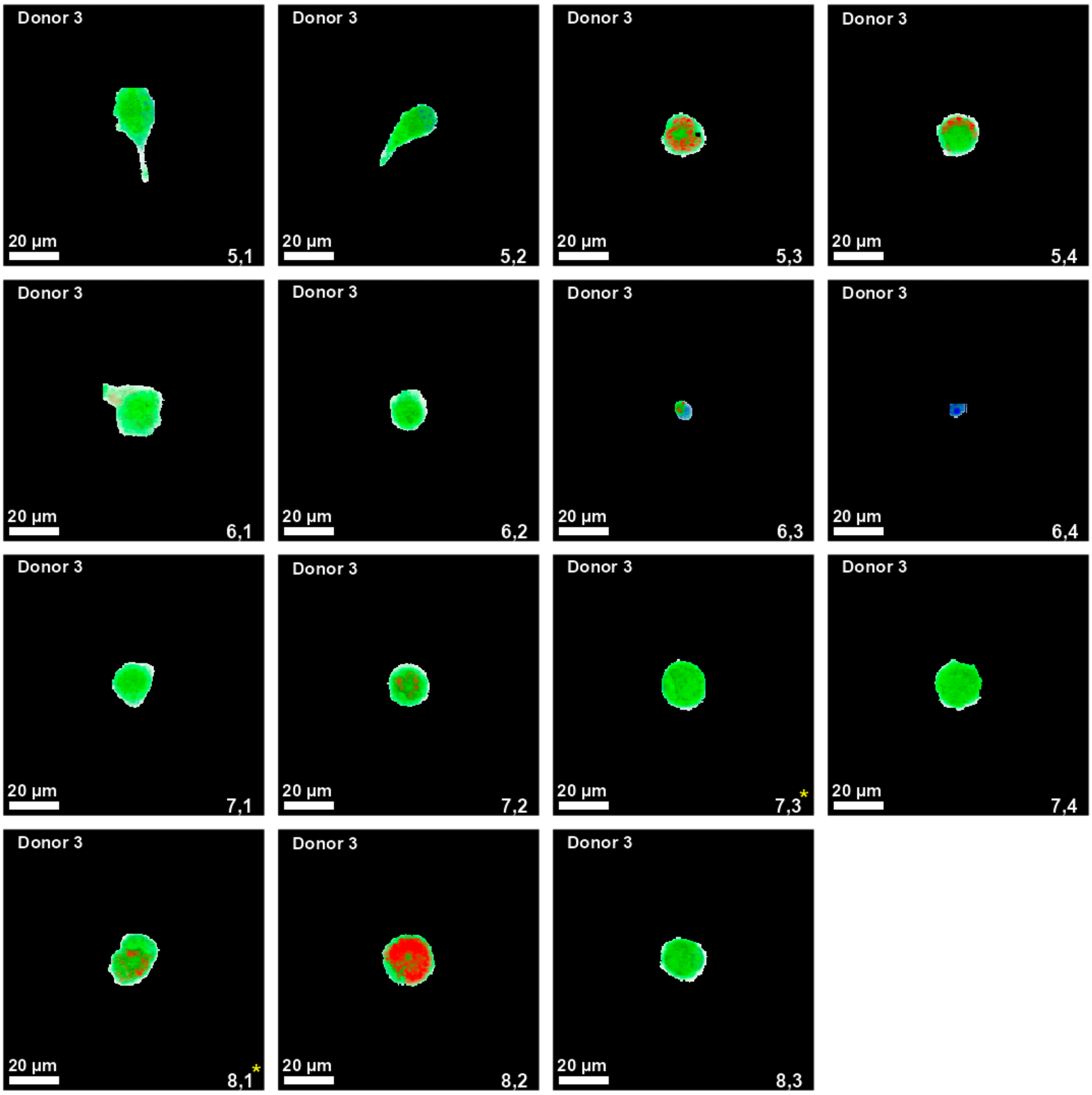

Supplemental Figure S4-C. False-colour Raman images of M1 macrophages. Colours: the colour of a pixel is assigned according to contribution of the corresponding group of endmembers shown in Fig. S7-A: red for lipids, green for cytoplasm proteins, blue for nucleus, and white for the cytoplasm/ cell environment (note: this endmember is depicted in black in Fig. S7-A). Scale: To make images comparable, each image is plotted in a square of 110 µm x100 µm area, independent of size of image scan. Scale bar is always 20 µm. Labels: Labels on the top-left indicate donor of the cell; bottom-right labels give a coordinate for easier reference. The images that were selected for finding endmembers have yellow asterisk near the coordinate label.

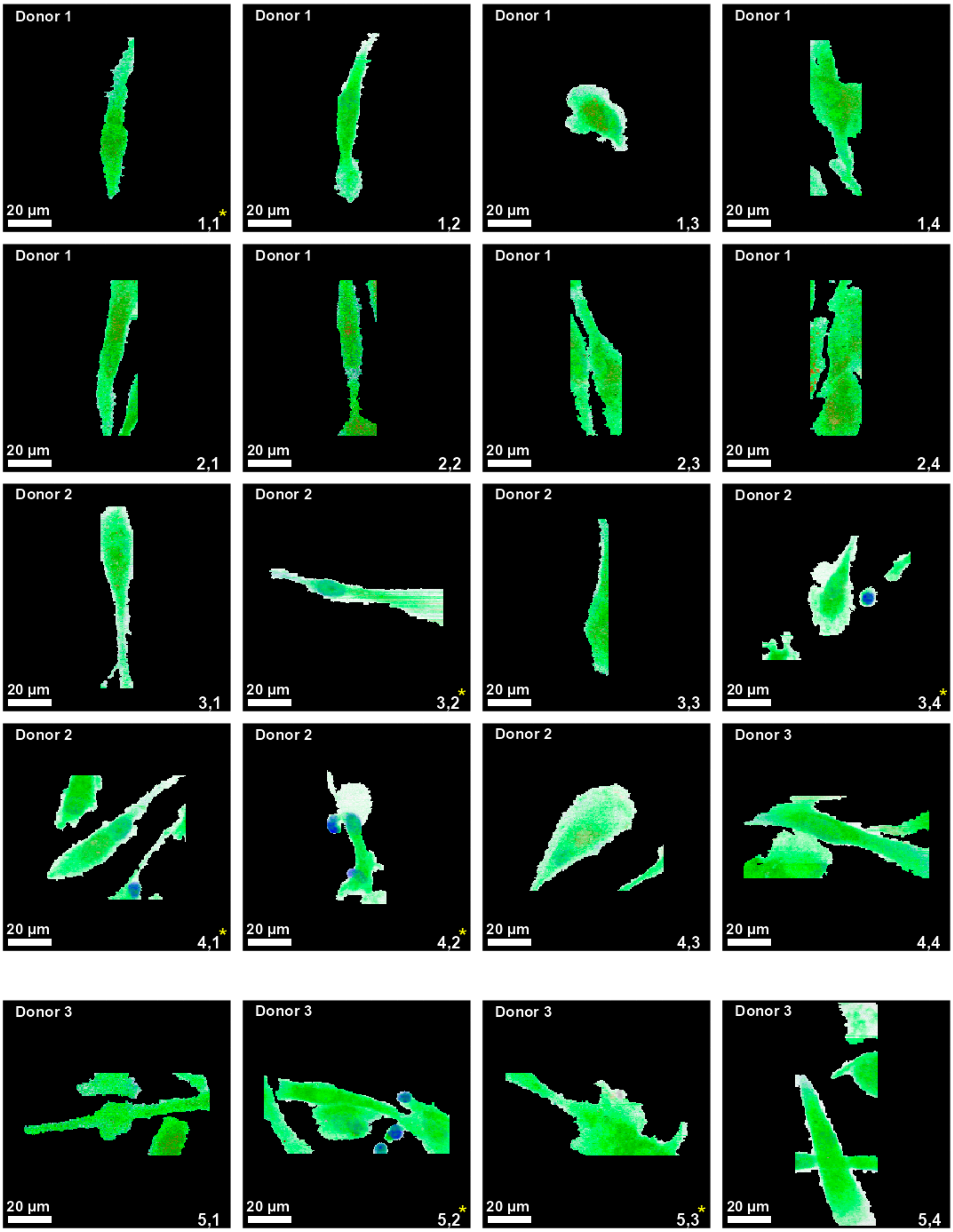

Supplemental Figure S4-D. False-colour Raman images of M2 macrophages. Colours: the colour of a pixel is assigned according to contribution of the corresponding group of endmembers shown in Fig. S7-A: red for lipids, green for cytoplasm proteins, blue for nucleus, and white for the cytoplasm/ cell environment (note: this endmember is depicted in black in Fig. S7-A). Scale: Each image is plotted in a square of 110 µm x100 µm area, independent of size of image scan. Scale bar is always 20 µm. Labels: Labels on the top-left indicate donor of the cell; bottom-right labels give a coordinate for easier reference. The images that were selected for finding endmembers have yellow asterisk near the coordinate label.

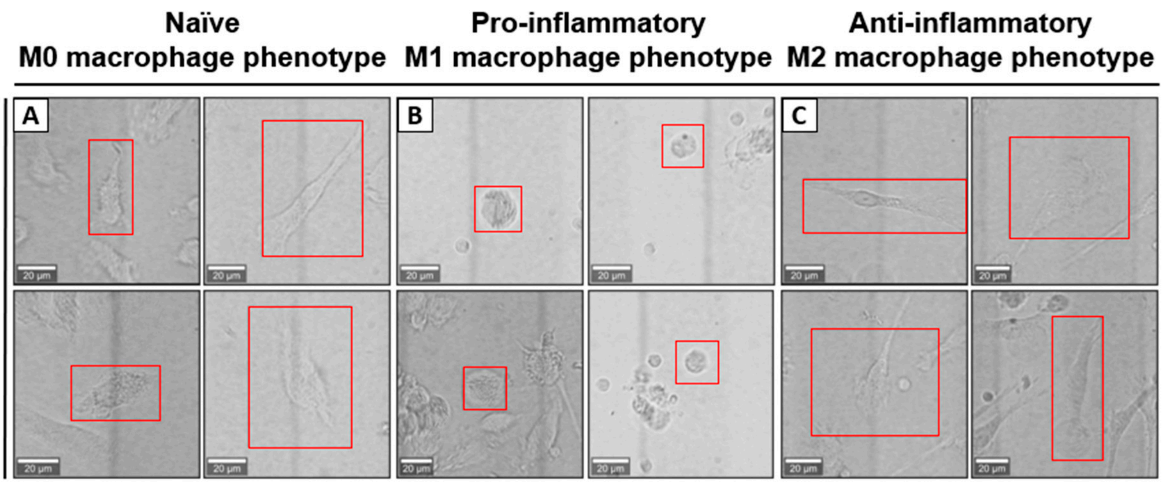

Supplemental Figure S4-E. Bright-field images of M0, M1 and M2 macrophages. Bright-field images shown here are from the cells depicted as false colour Raman images generated by N-FINDR analysis in Figure 2 (G, H, I), main manuscript. (A) M0 macrophages, (B) M1 macrophage and (C) M2 macrophages. The red squares indicate the region in which Raman imaging was performed. Scale bar is always 20 µm.

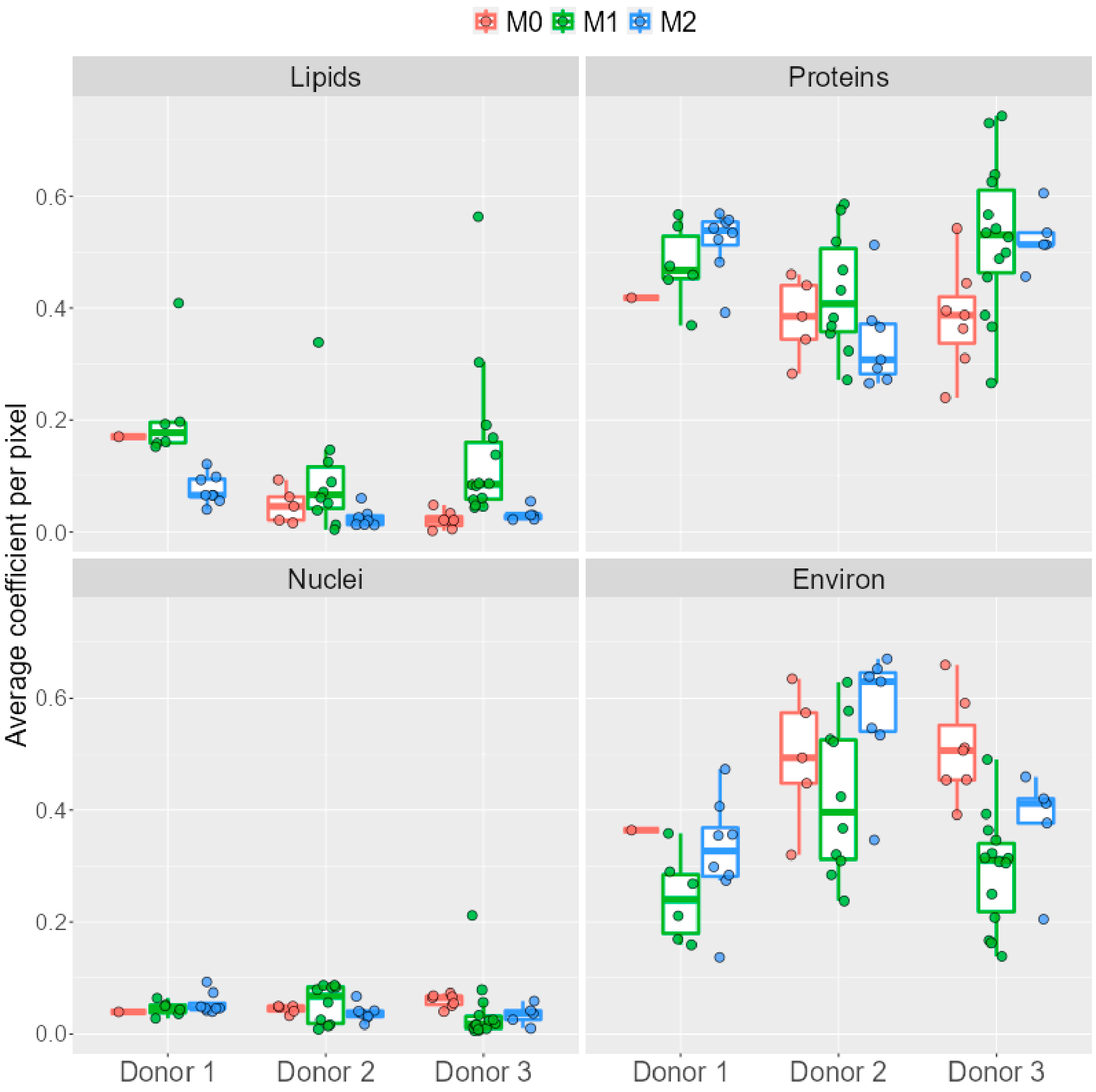

Supplemental Figure S5. Box-plots of average coefficients (contribution) per pixel split for each donor. A point represents the corresponding value of average coefficient per pixel for a single cell. Based on those values, box-plots were plot to represent the distribution of values: the box corresponds to the range from 25th (Q1) to 75th (Q3) percentile, the line inside the box – 50th percentile (i.e. Q2 or median), and the whiskers demonstrate the interquartile range from Q1 – 1.5*(Q3-Q1) to Q3 + 1.5*(Q3-Q1). The average, pro-inflammatory M1 macrophages have higher contribution of lipids (p-values: 0.0047 for Donor 1, 0.099 for Donor 2, and 0.00006 for Donor 3). In contrast to differences in protein contribution, the difference in lipids is persistent from donor to donor. p-values of Kruskal-Wallis test for lipids are 0.0047 for Donor 1, 0.099 for Donor 2, and 0.00006 for Donor 3.

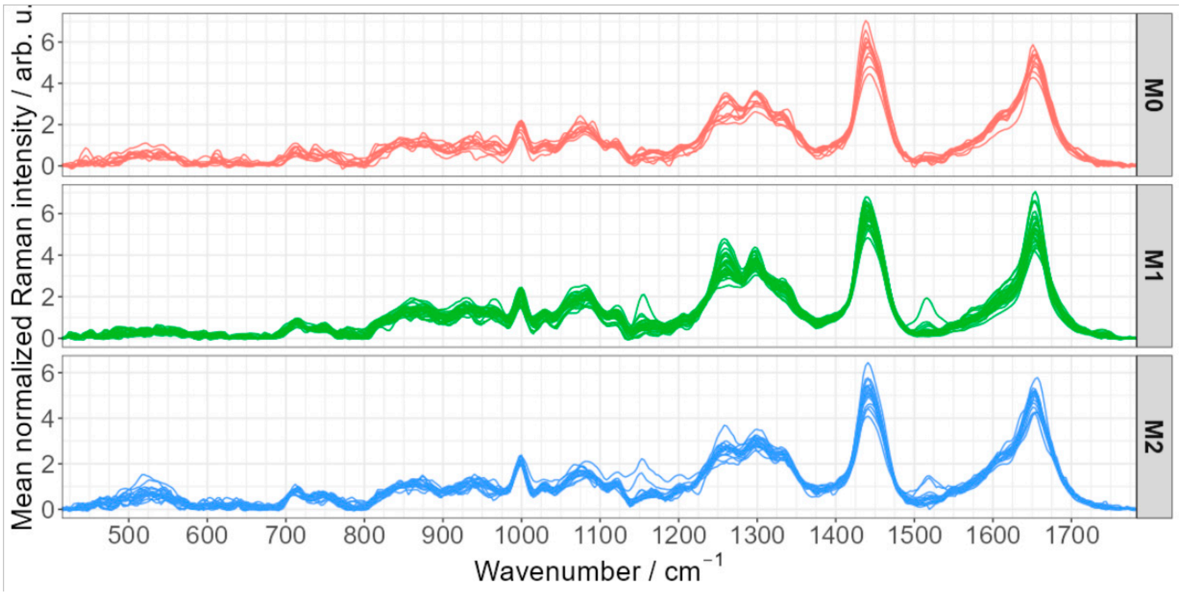

Supplemental Figure S6. Raman analysis of extracted lipid spectra.

Supplemental Figure S6-A. Averaged spectra of lipids in the fingerprint region extracted from Raman images of different macrophage phenotypes. One line represents the average spectrum of lipids from one cell (mean normalized). For extraction of lipid spectra from the unmixed N-FINDR images, all pixels (spectra) where total contribution of lipids was not less than 25% (i.e. N-FINDR coefficient for lipids ≥ 0.25), were used. Then the spectra were additionally filtered by k-means clustering since thresholding N-FINDR coefficients do not guarantee perfect match and, also, the threshold value of 0.25 was chosen to be low to capture more spectra.

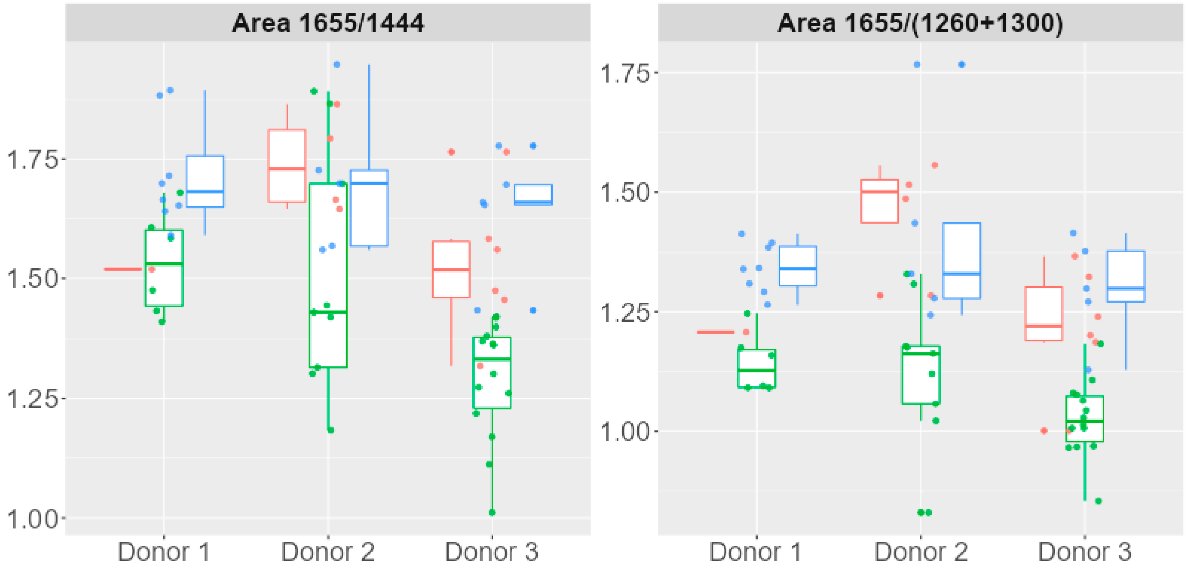

Supplemental Figure S6-B. Raman band ratios in extracted lipid spectra. Values are split by donor and colored by macrophage phenotype (red: M0, green M1, blue: M2). One line represents the average spectrum of lipids from one cell (mean normalized). For extraction of lipid spectra from the unmixed N-FINDR images, all pixels (spectra) where total contribution of lipids was not less than 25% (i.e. N-FINDR coefficient for lipids >= 0.25), were used. Then the spectra were additionally filtered by k-means clustering since thresholding N-FINDR coefficients do not guarantee perfect match and, also, the threshold value of 0.25 was chosen to be low to capture more spectra.

Supplemental Table S2. Overview of extracted lipid spectra. In total, 10,628 spectra were selected from 58 images (out of 183,596 spectra from 65 images). More details are provided in the table below

Group	Number of images selected				Number of spectra selected			
	Donor 1	Donor 2	Donor 3	∑	Donor 1	Donor 2	Donor 3	∑
M0	1	4	6	11	665	466	383	1,514
M1	6	9	14	29	2,490	824	2,752	6,066
M2	8	5	5	18	2,571	107	370	3,048
∑	15	18	25	**58**	5,726	1,397	3,505	**10,628**

On average 183 spectra were extracted per cell with 138 spectra of M0 cells, 209 from M1 cells and 169 from M2 cells.

Supplemental Table S3. Raman band ratios of lipid spectra in different macrophage phenotypes. Mean plus-minus standard deviation of each calculated Raman band intensity and Raman band ratio for the extracted lipid spectra shown in Supplemental Figure S6.

Raman band or ratio	M0	M1	M2
Intensity 1655 cm^{-1}	217.51 (±63.83)	412.00 (±133.50)	214.18 (±95.41)
Intensity 1444 cm^{-1}	227.90 (±63.84)	443.94 (±167.99)	213.48 (±102.36)
Intensity 1260 cm^{-1}	111.29 (±39.37)	252.65 (±114.52)	102.62 (±49.69)
Intensity 1300 cm^{-1}	123.27 (±35.22)	257.63 (±105.21)	111.13 (±48.66)
Area 1655/1444	1.60 (±0.16)	1.41 (±0.20)	1.69 (±0.13)
Area 1655/(1260+1300)	1.31 (±0.17)	1.08 (±0.12)	1.35 (±0.13)
Intensity 1655/1444	0.97 (±0.06)	0.96 (±0.11)	1.03 (±0.06)
Intensity 1300/1260	1.14 (±0.11)	1.05 (±0.10)	1.11 (±0.08)

3.2. *PCA-LDA models*

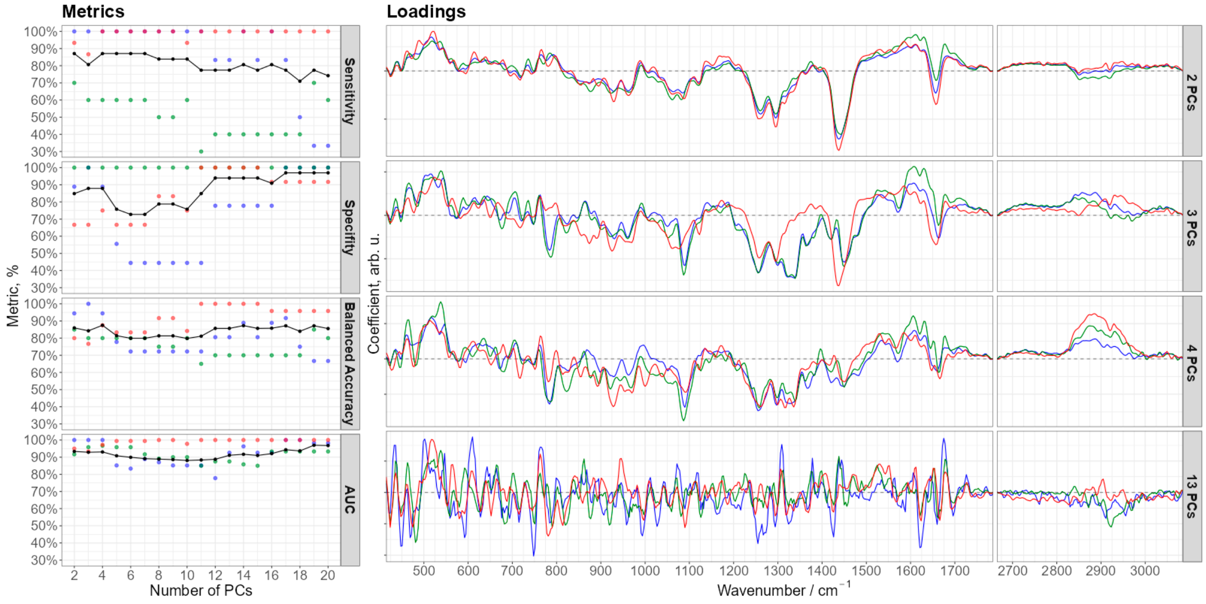

Supplemental Figure S7-A. Binary PCA-LDA model (trained on averaged spectra) identifying pro-inflammatory M1 macrophages*.* Colour codes the respective donor used in the test data set, i. e., left out in the training: blue: donor 1, green: donor 2, red: donor 3.

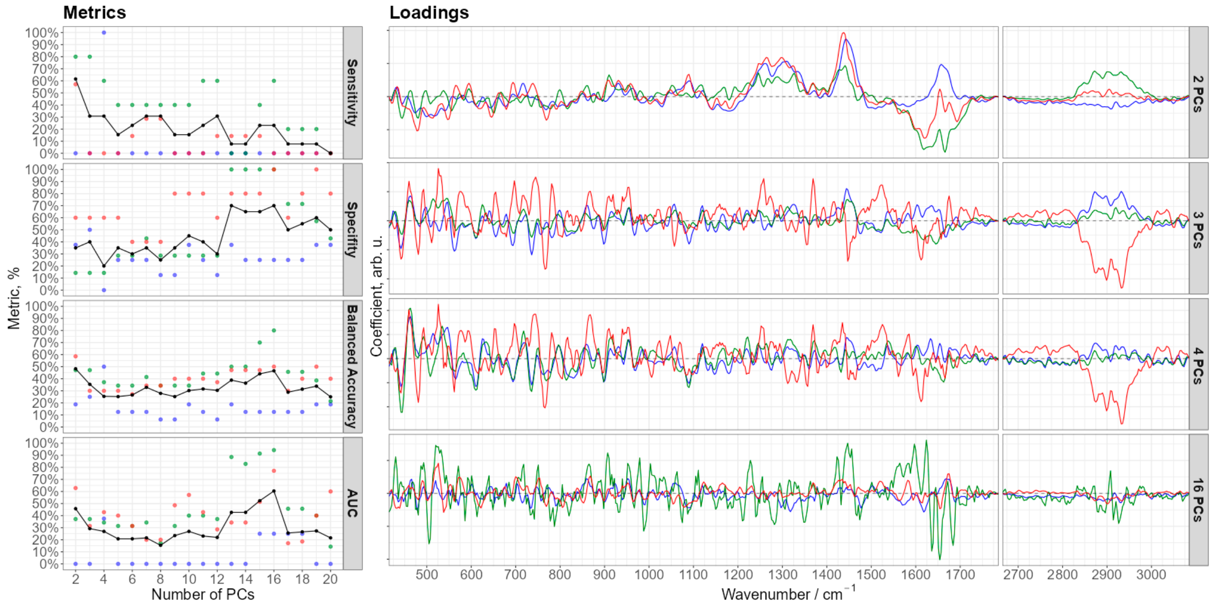

Supplemental Figure S7-B. Dependency of the performance metrics of the binary PCA-LDA model (trained on averaged spectra) discriminating between resting M0 and anti-inflammatory M2 macrophages on the number of principal components used*.* Colour codes the respective donor used in the test data set, i. e., left out in the training: blue: donor 1, green: donor 2, red: donor 3.

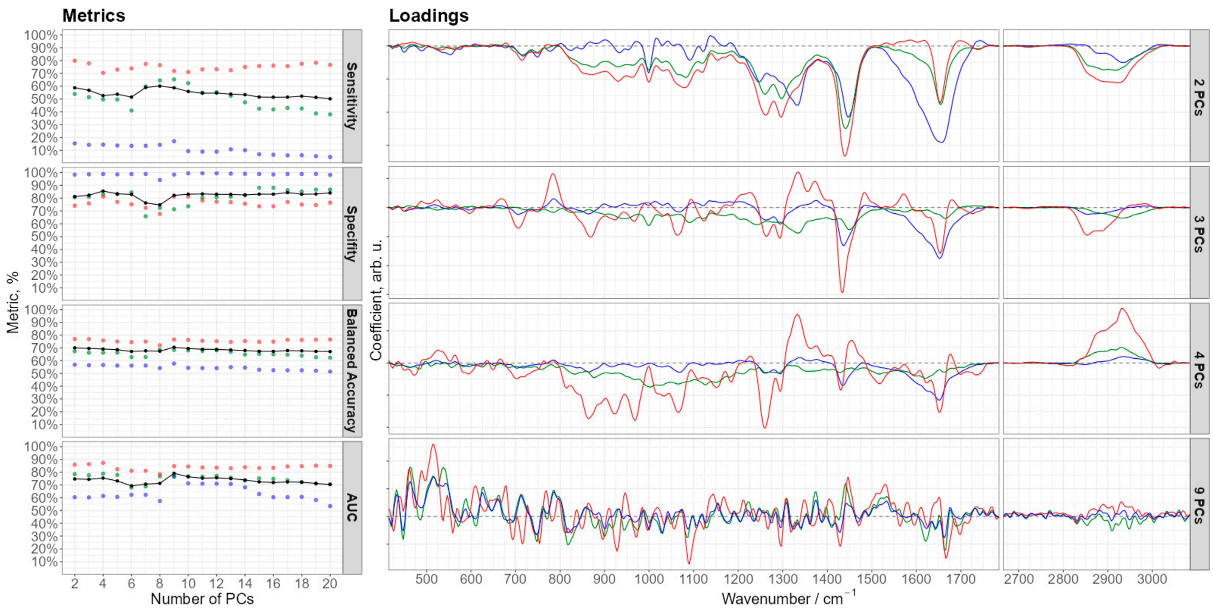

Supplemental Figure S7-C. Binary PCA-LDA model (trained on individual spectrum) identifying pro-inflammatory M1 macrophages.

Metrics: Dependency of the performance metrics on the number of principal components used. The image shows values of four performance metrics (from top to bottom: Sensitivity, Specificity, Balanced accuracy, and AUC) for different number of principal components used (from 2 to 20). The values are calculated using predictions on testing datasets of the leave-one donor-out cross-validation. For each number of principal components, four values are presented: coloured dots (blue, green, and red) represent predictions on the corresponding donor when it was in the testing set, while black dots represent values calculated on all testing predictions combined.

Loadings: Variation of coefficients for different number of principal components. The coloured lines (blue, green, and red) represent PCA-LDA coefficients when the corresponding donor was in the testing set. The image shows that increasing number of principal components also increases the variance of the model coefficients, i.e. makes predictions less stable.
